# Management of Eosinophilic Granuloma Occurring in the Appendicular Skeleton in Children

**DOI:** 10.4055/cios.2009.1.2.63

**Published:** 2009-05-26

**Authors:** Ilkyu Han, Eun Seok Suh, Sang-Hoon Lee, Hwan Seong Cho, Joo Han Oh, Han-Soo Kim

**Affiliations:** Department of Orthopedic Surgery, Seoul National University Hospital, Seoul, Korea.

**Keywords:** Langerhans cell histiocytosis, Indomethacin, Children

## Abstract

**Background:**

We compared indomethacin therapy with the more aggressive approaches of anti-cancer chemotherapy and surgery in the treatment of isolated Langerhans cell histiocytosis (LCH) of bone in children.

**Methods:**

Comparisons were made with respect to healing of the lesion without recurrence, time to radiological healing of the lesion, time to functional recovery, and complications related to treatment.

**Results:**

Complete radiologic healing of the lesion (mean, 15.3 months) and functional recovery (mean, 5.6 months) were observed in all patients treated with either approach. No significant differences were noted in the time to complete radiologic healing or the time to functional recovery between the two groups. There were no recurrences with either approach until the last follow-up (mean, 56 months). Complications were common with anti-cancer chemotherapy, but indomethacin was well-tolerated.

**Conclusions:**

Indomethacin seems to be effective for treating isolated LCH of bone in children. Hence, morbidities associated with aggressive treatment approaches such as anti-cancer chemotherapy or surgery can be avoided.

Langerhans cell histiocytosis (LCH) encompasses a group of histologically similar disorders, characterized by infiltration of tissues by cells of monocyte-macrophage lineage.^1^,2) The clinical forms of LCH vary widely, from generalized and fulminant forms to localized and curable forms.^3^,4) Isolated LCH of bone, also known as eosinophilic granuloma, is one of the mildest forms of the disease, with satisfactory results being reported especially in children.5) Many different approaches have been used for the treatment of isolated LCH of bone in children,^6^,7) from modest approaches such as simple observation and steroid injections8) to aggressive approaches such as surgery, anti-cancer chemotherapy, and radiation therapy.^5^,^9^,10) A treatment approach that carries a lower chance of complications while ensuring a successful cure is desirable in this cohort of patients.

Indomethacin is a nonsteroidal anti-inflammatory drug that inhibits cyclo-oxygenase, thereby blocking the arachidonic acid-prostaglandin pathway. Prostaglandins (PG) have been implicated in the pathogenesis of LCH. Purified LCH cells from bony lesions produce interleukin 1 and PGE2 in vitro,11) and LCH cells in cases of disseminated LCH have been shown to produce PGD2 and thromboxane.12) The use of indomethacin in treating various forms of LCH has been previously reported.^13^,14) However, no study comparing indomethacin with other treatment modalities in isolated LCH of bone has been reported.

We first sought to determine if indomethacin therapy induces complete healing in isolated LCH of bone in children. We also sought to determine if indomethacin therapy is comparable to anti-cancer chemotherapy or surgery with respect to the time to radiological healing of the lesion, the time to functional recovery, and complications related to treatment.

## METHODS

We reviewed the records of 85 patients with histologically proven LCH who had been managed in the authors' institution since 1988. Thirty-three patients who met the following criteria were included in the study: 1) histological diagnosis of LCH; 2) single bone involvement excluding lesions of skull, spine, or facial bones; 3) under 15 years of age; 4) no extraskeletal involvement. There were 18 boys and 15 girls, and the average age at the time of diagnosis was 6.7 years (range, 1 to 14 years). Eight different bones were involved (femur 8, clavicle 5, tibia 6, humerus 4, pelvis 4, scapula 2, fibula 2, radius 2). Most patients presented with pain or limping.

Biopsies were performed in all patients (incisional in 21, excisional in 8, and percutaneous in 4). The diagnosis of LCH was based on light microscopy findings of proliferation of histiocytes with eosinophilic cytoplasm, often associated with multinucleated giant cells. Immunochemical staining with CD1a or S-100 was performed in difficult-to-diagnose cases. Bone scans were performed to identify possible involvement of multiple bones. No patient had multiple osseous lesions or extraskeletal involvement.

Most of the patients treated in the early period of this series were managed with anticancer chemotherapy or excisional surgery. Indomethacin, a non-steroidal anti-inflammatory drug (NSAID), has been used in this group of patients at our institution since the mid-1990s. Therefore, patients in the current series were categorized into two groups: an aggressive treatment group (12 patients) in which patients were treated with excisional surgery, anti-cancer chemotherapy, or a combination of both; and a modest treatment group (21 patients) in which patients were treated with indomethacin. There were no noteworthy differences in age, gender ratio, or lesion locations between the aggressive treatment group and the modest treatment group (Table 1).

Four patients in the aggressive treatment group were treated with anti-cancer chemotherapy, 5 patients were treated with surgery, and 3 patients were treated with surgery followed by chemotherapy. Excisional curettage of the lesion and bone grafting were performed in 3 patients. Internal fixation was not performed in any patient. The anti-cancer chemotherapy regimen was as follows: 12 weeks of induction chemotherapy consisting of intravenous vinblastine (starting dose 0.15 mg/kg/week, weekly dose escalation of 0.5 mg), oral methotrexate (10 mg/m^2^/week), and oral cyclophosphamide (100 mg/m^2^/week), followed by maintenance chemotherapy consisting of oral 6-mercaptopurine (50 mg/m^2^/day), methotrexate (10 mg/m^2^/week), and cyclophosphamide (100 mg/m^2^/week). Maintenance chemotherapy was continued for one year unless side effects prevented completion. Patients were followed up weekly during the induction period and monthly during the maintenance period with complete blood counts and plain radiographs of their respective lesions. Bone scans were examined every 2-3 months during the treatment period. The average total duration of chemotherapy was 13.1 months (range, 9 to 15 months).

In the modest treatment group, indomethacin was used in 21 patients. A daily dose of 1-2.5 mg/kg of indomethacin was administered for an average of 7.7 months (range, 5 to 12 months). Patients were followed up with monthly plain radiographs, as well as bone scans every 2-3 months, during the course of treatment. Indomethacin was discontinued based upon the complete relief of symptoms and definitive progressive healing of the lesions on serial bone scans and radiographs.

Patients were followed up with plain radiographs and bone scans every 2-3 months after the completion of treatment. Healing of the lesions was determined using the radiological criteria of the Children's Cancer Study Group.15) A complete response was defined as complete radiographic disappearance of the lesion. A partial response was defined as at least 50 percent reduction in the lesion size and no appearance of new lesions. No response was defined as no disease improvement, a recurrence, or the development of a new systemic or osseous lesion. Functional recovery was based on the patient's return to a pain-free state with normal use of the affected part.5) "Pain-free" means the patient did not complain of any subjective pain, and "normal use of the affected part" means the motor power and range of motion of the affected part were the same as those in the unaffected part. The average follow-up duration was 56 months (range, 12 to 192 months).

All statistical analysis was performed with SPSS soft ware ver. 12.0 (SPSS Inc., Chicago, IL, USA). Student's t-test was used to compare the time to radiological and functional recovery between the aggressive treatment group and the modest treatment group. *p* values less than 0.05 were considered statistically significant.

## RESULTS

**Fig. 1:**
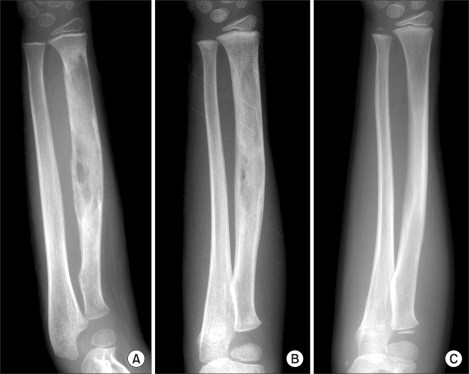
An illustrative case of isolated LCH of radius in a 5-year-old boy treated with indomethacin. (A) A destructive lesion of the radius is seen. (B) Radiographs made 3 months after treatment shows progressive healing of the lesion. (C) Radiographs made 15 months after treatment demonstrates the complete disappearance of the lesion and remodeling of the bone.

Complete radiologic healing was observed in all patients in an average of 15.3 months (range, 5 to 36 months) (Fig. 1). Complete functional recovery and symptomatic relief were achieved in an average of 5.6 months (range, 1 to 19 months). There were no significant differences between the two groups with respect to the time to complete radiologic response or the time required to return to a pain-free state with normal use of the affected part (Table 2). No patients had recurrence or developed new systemic or osseous lesions until the last follow-up visit. No patients developed new symptoms, such as pain or limping during follow-up. Furthermore, we noted no new lesions in x-rays or bone scans.

Complications related to anti-cancer chemotherapy were common, with all 7 patients who underwent chemotherapy experiencing one or more complications. Neutropenia that necessitated cessation of treatment was observed in 6 patients, all of whom recovered after 1 to 2 weeks. Oral mucositis and epigastric pain were observed in 2 patients each. Vomiting was observed in 2 patients. Indomethacin was well-tolerated in all patients, except for one, who experienced temporary dizziness that resolved spontaneously. Pathologic fractures were observed in two patients, both of whom had lesions of the femur. One patient who was two months into indomethacin treatment had a fracture of the femoral shaft and was treated successfully with closed reduction and external fixation. Another patient suffered from a fracture of the femoral shaft 4 months after excisional curettage and was treated successfully with a hip spica cast.

## DISCUSSION

LCH entails a disease spectrum including systemic entities such as Hand-Schuller-Christian disease and Letter-Siwe disease, and treatment modalities used for isolated LCH of bone have been variable. Satisfactory results have been reported for various treatment modalities, giving rise to the hypothesis that isolated LCH of bone is a benign pathophysiologic process. Eosinophilic granuloma of the spine is known to resolve spontaneously with time in children, and surgery is usually not required.^16^,17) In view of the possibility of spontaneous regression, less invasive forms of treatment with lower rates of complications are desirable. Our results support this approach. This study compared the results obtained with modest treatment modalities-namely indomethacin-with those obtained with aggressive treatment modalities, such as chemotherapy or surgery. All patients achieved complete bony healing and functional recovery in a similar time period. There were no local recurrences at the final follow-up visit.

The precise mechanism of action of indomethacin in LCH has not been determined. In vitro studies have demonstrated production of prostaglandins in isolated LCH cells,^11^,12) providing the basis for the use of indomethacin in a previous study.13) Indomethacin is a nonsteroidal anti-inflammatory drug that inhibits cyclo-oxygenase, thereby blocking the arachidonic acid-prostaglandin pathway. Successful use of intralesional steroids has been reported in the setting of localized LCH,^18^,19) and the mechanism of action is presumed to be inhibition of prostaglandins.20) Whether indomethacin influences the disease process or simply acts as an analgesic remains unclear.

Munn et al.13) reported the initial experience with indomethacin in 10 LCH patients. Six patients had single-system bone disease, and 4 had multi-system disease involving the bony skeleton and other organs. All 6 patients with single-system bone disease had complete responses to treatment, defined as complete relief of pain.

Two pathological fractures occurred in patients with femur lesions, one each in the modest treatment group and in the aggressive treatment group. The small number of pathologic fractures makes comparison of the incidence of pathologic fractures between the two groups difficult. However, appropriate protective measures should be taken if the lesion is located in a weight-bearing area and poses a risk of pathologic fracture.9)

There are several limitations to this study. First, the study was performed in a relatively small, nonrandomized, retrospectively selected group of patients. Prospective comparison in a larger number of patients is desirable to establish the role of indomethacin in isolated LCH of bone, although the infrequency of the condition would make such a study difficult to perform. Secondly, the follow-up period was significantly shorter in the group treated with indomethacin. Long-term effects of indomethacin in children could be clarified with longer follow-up.

In conclusion, indomethacin seems to be effective for treating isolated LCH of bone in children. Morbidities associated with aggressive treatment approaches such as anticancer-chemotherapy or surgery may be avoided through the use of indomethacin instead.

## Figures and Tables

**Table 1:**
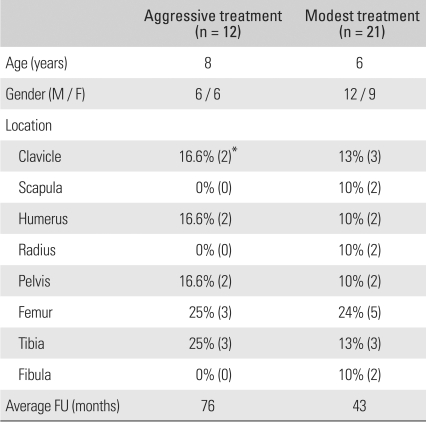
Comparison of Clinical Presentation of the Two Groups

^*^Numbers in the parentheses represent the number of cases.

**Table 2:**
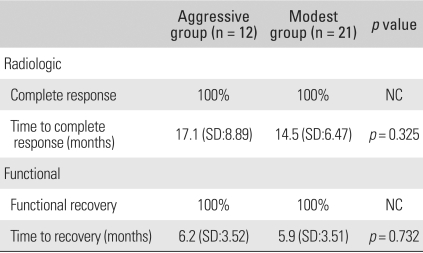
Radiological and Functional Results of the Two Groups

NC: Not calculable.
